# Pharmacological Interactions of Epinephrine at Concentrations Used in Dental Anesthesiology: An Updated Narrative Review

**DOI:** 10.3390/reports8040224

**Published:** 2025-10-31

**Authors:** Maria Aikaterini Saraga, Ioannis Fotopoulos, Vasileios Zisis, Athanasios Poulopoulos, Nikolaos Dabarakis, Theodoros Lillis

**Affiliations:** 1 Department of Dentoalveolar Surgery, Surgical Implantology and Radiology, School of Dentistry, Aristotle University of Thessaloniki, 54124 Thessaloniki, Greece; 2 Department of Oral Medicine/Pathology, School of Dentistry, Aristotle University of Thessaloniki, 54124 Thessaloniki, Greece; akpoul@dent.auth.gr; 3 Department of Dentistry (Oral Medicine-Oral Pathology), School of Dentistry, European University, Diogenous Street 6, 2404 Nicosia, Cyprus

**Keywords:** epinephrine, dental anesthesia, pharmacological interactions, clinical studies, classification criteria

## Abstract

The widespread use of pharmaceutical agents highlights the importance of identifying potential pharmacological interactions with epinephrine, the most frequently used vasoconstrictor in dental practice. Dentists must be aware of possible risks in order to adjust anesthetic protocols, when necessary. The principal aim is to prevent complications and ensure patient safety. This review analyzes clinical data from the international literature on pharmacological interactions involving low-dose epinephrine, corresponding to the doses typically used in dental procedures. These interactions are subsequently classified according to their severity and documentation level, based on the criteria of the UpToDate Lexidrug platform. In addition, management strategies are proposed to guide dental practitioners in clinical decision-making. A literature search was conducted in PubMed, Scopus, Web of Science, and Cochrane Library databases, using specific keywords. In total, 24 studies met the inclusion criteria, with the earliest published in 1968 and the most recent in 2022. Nine pharmacological categories were identified and presented in tables. The dosage of epinephrine plays a key role in the likelihood of pharmacological interactions, which appear to be less frequent at low concentrations typically used in dentistry. However, patient-specific factors, such as overall health status, should also be carefully considered during clinical assessment.

## 1. Introduction

In recent years, advances in medicine and pharmacology have significantly improved quality of life and increased life expectancy [1]. At the same time, the growing prevalence of chronic diseases and easier access to medications have contributed to the widespread phenomenon of polypharmacy [2]. Additionally, there has been a notable rise in the prescription of medications to younger individuals—even children—for pathological or psychiatric conditions that were not previously treated pharmacologically, a trend internationally known as “pharmaceuticalization” [3,4,5,6,7]. In this context, dentists must take thorough medical histories for patients of all ages, as existing pharmacological treatments may interact with drugs used during dental procedures—particularly vasoconstrictors included in local anesthetic solutions [8,9].

Despite potential concerns, the use of vasoconstrictors remains indispensable in dental anesthesia due to their clear clinical benefits. Local anesthetics like lidocaine have vasodilatory effects, leading to faster absorption, reduced duration, and compromised pain control [10]. Adding a vasoconstrictor counteracts this, by prolonging anesthesia, improving hemostasis, and reducing systemic toxicity, through slower absorption. It also enables lower doses and fewer reinjections [11,12,13,14].

Epinephrine, also known as adrenaline, is the most widely used vasoconstrictor in dental anesthetic solutions [15]. When administered exogenously, epinephrine acts as a peripheral agonist of adrenergic receptors, without affecting the central nervous system, as it does not cross the blood–brain barrier. Its action is limited to the postsynaptic adrenergic receptors α_1_, α_2_, β_1_, and β_2_ [16,17]. Specifically, epinephrine is a sympathomimetic catecholamine that exerts its pharmacological effects by stimulating both α- and β-adrenergic receptors of the autonomic nervous system [12,16,17,18]. Activation of α_1_-adrenergic receptors causes vasoconstriction in the mucosa, skin, and some visceral organs, potentially leading to an increase in blood pressure [16,17,18]. Stimulation of α_2_-receptors also contributes to peripheral vasoconstriction, enhancing the effect of α_1_-receptors [16,17,18]. Epinephrine also binds to β_1_-receptors in the heart, increasing heart rate and myocardial contractility, which may indirectly elevate systolic blood pressure [12,16,17,18]. Finally, by activating β_2_-receptors, it induces bronchodilation and vasodilation in the skeletal muscle, which can lead to a decrease in diastolic blood pressure [16,17]. Generally, β-adrenergic effects tend to be more systemic, influencing multiple organs and systems, whereas α-adrenergic effects are more localized in the periphery, with limited systemic action [12,13,14,18,19,20,21,22,23]. It is also worth noting that epinephrine’s systemic effects are very short-lived, due to its rapid plasma half-life, which is less than one minute [12,21], and its swift metabolism by catechol-O-methyltransferase (COMT) in the blood, liver, lungs, and other tissues—typically within 10 min [12].

In dental anesthesia, epinephrine acts as a highly effective local vasoconstrictor by targeting α_1_-adrenergic receptors in small blood vessels of the oral mucosa, submucosa, and periodontium, where these receptors predominate [13,19,23,24]. Epinephrine has become the vasoconstrictor of choice in dentistry, replacing agents like norepinephrine due to its superior safety and pharmacological profile [25]. Its α_1_-adrenergic action causes vasoconstriction, while β_2_ activity induces vasodilation in skeletal muscle, balancing vascular effects and supporting hemodynamic stability [12,25]. Unlike norepinephrine, which lacks β_2_ action, epinephrine causes less prolonged vasoconstriction and has a faster onset with shorter systemic effects, making it better tolerated [14,25].

In dentistry, epinephrine concentrations of 1:80,000, 1:100,000, and 1:200,000 are commonly used [26]. The 1:50,000 concentration is now limited to procedures requiring greater hemostasis due to higher cardiovascular risk. ADA/AHA guidelines recommend 1:100,000 or 1:200,000 for routine use [12,27]. The maximum dose per appointment is 0.2 mg for healthy adults and 0.04 mg for cardiac patients [11,27].

Epinephrine’s receptor affinity is dose-dependent. At low doses, it primarily stimulates β-receptors, while higher doses activate α-receptors. Thus, its effects on blood pressure and heart rate vary by dose [18]. In dentistry, the low doses used may cause β_2_-mediated vasodilation in skeletal muscle, reducing diastolic pressure, while β_1_ stimulation raises systolic pressure and heart rate, with minimal impact on mean arterial pressure [24]. However, systemic effects such as β_1_-induced tachycardia may be undesirable in patients with cardiovascular disease. The risk of systemic responses depends on factors such as intravascular injection, vasoconstrictor type and dose, total volume, and the patient’s health status [19,23]. Elevated plasma epinephrine levels—whether from overdose or accidental injection—can enhance interactions, particularly with drugs affecting the sympathetic or cardiovascular systems [25]. Recognizing these interactions is key to safe dental practice.

Drug interactions occur when the effect of one drug is altered by the simultaneous presence of another. These can be beneficial or harmful [28]. They are generally classified as pharmacokinetic—where one drug affects the absorption, distribution, metabolism, or excretion of another—or pharmacodynamic, where the interaction occurs at the action site, without changing drug concentration. Pharmacodynamic interactions may be additive, synergistic, or antagonistic, depending on whether the combined effect equals, exceeds, or diminishes the individual effects [21,28,29]. This section focuses on drug interactions involving epinephrine, which primarily occur at the adrenergic neuron. Norepinephrine, the main endogenous neurotransmitter, activates α_1_ and β_1_ receptors—targets also stimulated by exogenous epinephrine. Epinephrine in local anesthetics is metabolized by catechol-O-methyltransferase (COMT). Thus, interactions may arise from drugs that either affect receptor activity (pharmacodynamic) or inhibit COMT-mediated breakdown (pharmacokinetic) [20,25].

While interactions with high epinephrine doses are well documented, those involving low concentrations used in dental anesthesia are less clearly defined, limiting firm clinical conclusions. This study aims to explore potential pharmacological interactions that may arise from the co-administration of low-dose epinephrine (up to 54–67.5 μg), as typically used in dental local anesthetic solutions, with specific classes of medications. It also aims to classify these interactions according to standardized criteria defined by the evidence-based UpToDate Lexidrug database, in order to promote the safe use of vasoconstrictors in dentistry and ensure patient safety.

## 2. Material and Method

A comprehensive literature search was conducted in PubMed, Scopus, Web of Science, and Cochrane Library databases from March 2022 to 29 September 2024, aiming to identify studies published between 1965 and 2024, focusing on the topic: “Pharmacological interactions of epinephrine at concentrations used in dental anesthesiology.” The search strategy was developed using keywords related to epinephrine, drug interactions, route of administration, and study design. These terms were combined using the Boolean operators AND and OR. Quotation marks (“ ”) were used to identify specific key phrases, while truncation with an asterisk (*) was applied to capture different word endings. The final search string used in the databases was as follows:

(epinephrine OR adrenaline) AND (“drug interact *” OR “drug agonism” OR “drug antagonism” OR “drug synerg *” OR “pharmac * interact *” OR “drug-drug interact *” OR “medica * interact *” OR interact * OR potential *) AND (infusion * OR anesthe * OR infiltration * OR injection * OR administration * OR intravenous OR exogenous) AND (trial OR study OR experiment * OR “case report *”).

**Figure 1:**
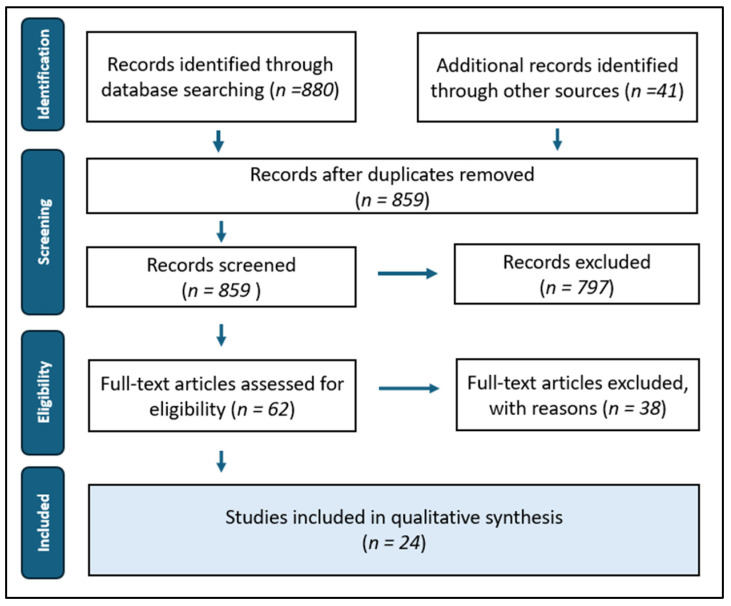
Study selection process.

Additionally, filters were used to limit results to specific article types, such as clinical studies in humans and animals, as well as case reports. Only English-language publications in the medical field were included, excluding unrelated disciplines such as mathematical sciences. Figure 1 illustrates the study selection process.

Following the database search across PubMed, Scopus, Web of Science, and Cochrane Library, and the application of appropriate filters, a total of 880 articles were initially retrieved, plus 41 from other sources. After removing duplicates via Mendeley software, 859 unique records remained for screening. A first-level screening excluded 797 articles based on predefined criteria, including irrelevance to the topic, reviews, editorials, non-clinical studies, and alternative administration routes (e.g., intraocular, epidural). Sixty-two articles remained, involving clinical studies or case reports in humans or animals where exogenous epinephrine was administered (locally or intravenously) and linked to pharmacological interactions.

Among the 62 studies, the key inclusion criterion was epinephrine dosage: only studies using up to 54–67.5 μg were included. This corresponds to a maximum of three cartridges of local anesthetic at concentrations commonly used in dentistry, namely 1:80,000 (22.5 μg/cartridge) and 1:100,000 (18 μg/cartridge), respectively. This threshold was applied to studies with intravenous epinephrine to simulate accidental intravascular injection during nerve block anesthesia (e.g., inferior alveolar block). The 3-cartridge limit reflects clinical reality, as inadvertent injection of higher doses during routine dental procedures is unlikely. Although higher doses may be acceptable in healthy patients during infiltration anesthesia, a uniform limit was applied across all techniques to ensure methodological consistency and maintain a homogeneous dosing framework, enabling reliable and comparable data analysis. This approach ensures that the assessment of pharmacological safety reflects realistic, routine clinical scenarios in dental practice. As a result, studies involving higher epinephrine doses were excluded. From the 24 studies that met the inclusion criteria, relevant data were extracted for comparative analysis.

The second part of this review aimed to classify all pharmacological interactions identified in the 24 included studies, based on the evaluation criteria established by the internationally recognized pharmacology platform UpToDate Lexidrug (formerly Lexicomp). These criteria assess each drug interaction according to its severity, level of documentation, and estimated risk—the latter determining the recommended clinical management strategy, as outlined in Table 1, Table 2 and Table 3, respectively. These Table 1, Table 2 and Table 3 s were created using the classification framework of the UpToDate Lexidrug database [30].

## 3. Results

### 3.1. Results of Studies on Low-Dose Epinephrine Interactions

Table 4, Table 5 and Table 6 summarize key data from the included studies—human trials, animal experiments, and case reports, respectively. Each table lists the sample size, participant health status, interacting drug, duration of use, epinephrine dose and route, and observed outcomes. The studies are presented in alphabetical order according to the pharmacological category examined, and in chronological order within each category. To ensure comparability, epinephrine doses are reported in micrograms (μg). As the review set an upper limit of 67.5 μg (equal to three dental cartridges), studies exceeding this dose were excluded. In studies where epinephrine was administered in gradually increasing doses, only data up to the 67.5 μg limit were included; results beyond this dose were excluded. For studies reporting epinephrine in μg/kg, total dose was calculated using a 70 kg adult reference weight, unless specified otherwise. Intraoral epinephrine administration was performed with standard precautions (e.g., slow injection, negative aspiration), as reported in the studies. Clinical manifestations resulting from hemodynamic changes are noted in the tables, when observed. Significant findings were defined as statistically supported changes in measured parameters, based on each study’s analytical methods.

A total of nine drug classes were evaluated for potential interactions with low-dose epinephrine, as used in dental procedures. These, listed in alphabetical order, included: antipsychotics, b-blockers, calcium channel blockers, catechol-O-methyltransferase (COMT) inhibitors, cocaine, digitalis glycosides, diuretics, monoamine oxidase inhibitors (MAOIs) and tricyclic antidepressants. Also, Table 7 includes examples linking the generic drugs to their respective commercial (brand) names.

Regarding psychotropic drugs interacting with epinephrine, literature data focused on antipsychotics, monoamine oxidase inhibitors (MAOIs) and tricyclic antidepressants. Antipsychotics were examined [8,31,49], but no interactions with low-dose epinephrine were reported—regardless of the route of administration (intravenous or local infiltration) or the duration of antipsychotic use (single dose or long-term). Three studies investigated the interaction between monoamine oxidase inhibitors (MAOIs) and low-dose epinephrine [8,47,51]. One study reported moderate but non-significant increases in heart rate and decreases in diastolic blood pressure, suggesting a 2- to 4-fold potentiation of epinephrine’s effect [47]. In contrast, pargyline was found to have no significant impact on the arrhythmia threshold [51], while phenelzine did not alter blood pressure or heart rate [8]. Finally, the interaction between tricyclic antidepressants and epinephrine was explored in four studies [8,48,51,52]. While some studies [8,52] reported no significant hemodynamic changes, others [48,51] noted minor, not significant effects. One study observed slight increases in blood pressure and heart rate, even at low epinephrine doses, indicating enhanced sensitivity to epinephrine [48]. Likewise, another study [51] found that tricyclic use lowered the threshold for arrhythmia induction by epinephrine—though this was not evident at the low doses evaluated in this review.

Concerning the interaction between β-blockers and epinephrine, studies involving local infiltration found no significant changes in blood pressure or heart rate in patients on non-selective β-blockers [31,39,40,41]. In contrast, intravenous administration at similar doses [32,35,36] caused significant hypertension, and bradycardia, with clinical symptoms in one study [36], including arrhythmia and chest tightness. Other studies showed that epinephrine led to greater increases in blood pressure and reductions in heart rate in patients on non-selective β-blockers—regardless of administration route—compared to placebo or other antihypertensives [34,38,41]. Some also reported more pronounced hemodynamic effects in patients on non-selective vs. selective β-blockers [32,34,41]. Prolonged anesthesia duration was noted in one study [40], while more serious effects—such as reduced myocardial contractility [34,37], impaired diastolic function [38], increased myocardial oxygen demand [35], and decreased cardiac output or cardiac index [35,37]—were observed when epinephrine was combined with non-selective β-blockers. Increased total peripheral resistance was also reported [41]. However, most studies did not associate these changes with clinical symptoms. A noteworthy positive finding was reported in a study, which showed no epinephrine-induced drop in plasma potassium in chronic β-blocker users, contrary to the expected hypokalemic response [39]. Lastly, two case reports described severe reactions to low-dose epinephrine in long-term propranolol users: one patient experienced cardiac arrest but survived, and the other was successfully managed with immediate antihypertensive therapy [53].

Two clinical studies explored the interaction between calcium channel blockers and low-dose epinephrine [39,42]. In one, a mild but non-significant decrease in potassium was observed after epinephrine administration in patients receiving long-term nifedipine and β-blockers [39]. This effect was attributed to nifedipine, as β-blockers are known to counteract epinephrine-induced hypokalemia. The other found no potassium changes, but a significant, asymptomatic glucose reduction associated with diltiazem [42].

A study on catechol-o-methyltransferase inhibitors found that a single dose of entacapone with low-dose epinephrine caused no significant changes in blood pressure or heart rate [43]. Mild, non-significant increase in systolic blood pressure and heart rate, and a slight decrease in diastolic blood pressure were observed at 15 min (52.5 μg epinephrine), with no differences compared to placebo [43].

The only study examining the interaction between cocaine and low-dose epinephrine was conducted in sheep [50]. After prolonged cocaine exposure (15–18 days), following a pattern simulating human use, hemodynamic responses to epinephrine showed no difference compared to those without prior cocaine use, suggesting no interaction [50].

Cardiac glycosides, particularly digoxin, were investigated in a single clinical study, which reported a higher frequency of ECG changes—mainly tachycardia but not arrhythmias—in patients receiving digoxin, compared to 26 healthy controls [44]. Notably, these changes were not accompanied by clinical symptoms. Interestingly, a similar study without epinephrine administration reported more frequent ECG alterations, primarily involving arrhythmias.

Another drug category, diuretics—especially non potassium sparing diuretics—was examined [39,45,46]. Overall, no major hemodynamic changes were observed following epinephrine co-administration, except for one study [46], which reported a significant drop in diastolic blood pressure 10 min after local infiltration. All three studies found a greater reduction in plasma potassium levels when epinephrine was combined with non–potassium-sparing diuretics, compared to either agent alone. In two studies [39,46], 50% of participants developed hypokalemia (<3.1 mmol/L), with the lowest recorded value being 2.6 mmol/L. The third study [45] reported early mild reductions in potassium and magnesium levels at a low epinephrine dose (21 μg), though these changes were not statistically significant. No arrhythmias or clinical symptoms were reported in any of the studies.

### 3.2. Classification of Low-Dose Epinephrine Interactions Based on Lexidrug Criteria and Literature Data

Following a detailed analysis of the literature on potential interactions between low-dose epinephrine—as used in dental practice—and nine drug categories, this review aimed to classify these pharmacological interactions using the classification criteria (Table 1, Table 2 and Table 3) established by the internationally recognized UpToDate Lexidrug platform. In Table 8, each drug is evaluated for the severity of its interaction with low-dose epinephrine, as well as the reliability of the evidence supporting or disputing the interaction. Additionally, a risk rating—ranging from A to X—indicates the level of clinical attention or intervention required. This classification framework is intended to support healthcare professionals in making informed clinical decisions regarding the management of each potential drug interaction. All ratings are based on the data extracted from the studies included in this review.

## 4. Discussion

Epinephrine, in low concentrations, is widely used in dental anesthesiology. However, even at low doses, its co-administration with other pharmaceutical agents may lead to significant pharmacological interactions, which can have clinical implications, especially in patients with chronic conditions or a vulnerable cardiovascular system. This review examines and analyzes the potential interactions of epinephrine with nine different classes of drugs, as previously outlined. The following section evaluates the findings for each specific interaction, with emphasis on the possible mechanisms involved, their clinical relevance, and the potential implications for therapeutic decision-making.

Antipsychotics used to treat psychotic disorders are divided into typical (e.g., chlorpromazine) and atypical (e.g., risperidone) agents. Most typical antipsychotics are phenothiazine derivatives and are commonly referred to as “phenothiazines” [22,23,26,54,55]. They act as antagonists at dopaminergic, histaminergic, cholinergic, and α-adrenergic receptors, which accounts for various side effects, including orthostatic hypotension due to α-blockade [56]. The interaction between antipsychotics and epinephrine is mainly due to α1-adrenergic receptor blockade, which inhibits vasoconstriction and allows unopposed β2-mediated vasodilation. This may lead to hypotension, reflex tachycardia, and prolonged bleeding, especially at high antipsychotic doses [13,22,23,26,28,31,54,55,57,58,59,60,61,62,63,64,65]. In dentistry, this could also reduce anesthetic effectiveness [57,61]. Thioridazine, in particular, poses added risk due to its effects on cardiac conduction and ECG changes, which epinephrine may exacerbate, especially if injected intravascularly [23,54,58,64]. Chronic antipsychotic use may cause α1-receptor upregulation, reducing vasodilatory and hypotensive effects over time. Thus, early-stage patients are more prone to cardiovascular side effects. Binding affinity also varies; chlorpromazine and risperidone show stronger α1-blocking activity than others [31]. Animal [8,49] and human [31] studies showed no interaction between low-dose epinephrine and antipsychotics, whether used long-term or as a single dose. No dental case reports exist, and the only reported case [66] involved persistent hypotension after high-dose epinephrine in a patient on long-term clozapine, which resolved upon stopping epinephrine.

Beta-adrenergic blockers, used to treat hypertension, are divided into non-selective (block both β1 and β2 receptors) and selective (primarily block β1 receptors) [11,13,21,54,57,58,67]. Non-selective β-blockers inhibit epinephrine’s β-mediated effects—cardiac stimulation (β1) and vasodilation (β2)—resulting in unopposed α-adrenergic activity, which may cause severe hypertension and reflex bradycardia [11,13,22,24,26,29,54,55,57,58,59,67,68,69,70,71,72,73,74,75,76]. In contrast, selective β-blockers have a milder interaction with epinephrine [13,21,68,74]. This difference is supported by studies included in this review [32,34,41]. According to the reviewed studies, non-selective β-blockers are the only class of antihypertensives shown to cause significant hypertension and bradycardia when combined with low-dose epinephrine. Studies [34,38,41] comparing them with other treatments or placebo reported notable hemodynamic changes, suggesting a potentially harmful interaction. As a result of this interaction, various adverse effects of differing severity may occur. One clinical observation was prolonged pulpal and soft tissue anesthesia [40], attributed to excessive vasoconstriction. This effect may increase the risk of local ischemia and necrosis, especially in hypertensive patients with compromised vascular system. More serious, life-threatening complications included reduced myocardial contractility [34,37], diastolic dysfunction [38], increased cardiac pressure work [35], reduced cardiac output and index [35,37], and elevated peripheral resistance [35,37]. These effects result from β1-blockade preventing epinephrine’s positive inotropic action, shown by unchanged P/V ratios, reflecting no increase in contractility. Non-selective β-blockers may also suppress baseline myocardial function by blocking both exogenous epinephrine and endogenous sympathetic tone. Diastolic dysfunction further impairs ventricular filling, while increased oxygen demand adds to the risk of myocardial ischemia. Although no clinical symptoms were reported in these studies—likely due to testing in healthy individuals—such responses may become clinically significant in patients with cardiovascular disease. Only a few cases showed clinically evident symptoms. One study [36] reported arrhythmia-related symptoms and chest tightness. Two case reports [53] described severe hypertension and bradycardia: one involved cardiac arrest successfully managed with CPR, and the other resolved after antihypertensive treatment. Interestingly, a beneficial effect was noted in one study [39], where non-selective β-blockers prevented the epinephrine-induced drop in plasma potassium levels, likely by blocking β2-mediated hypokalemia. In six of ten clinical studies [35,36,37,38,40,47], β-blockers were given as a single dose. It was suggested [35,38] that the duration of β-blocker administration may influence outcomes, as chronic use reduces vasoconstriction via compensatory mechanisms, like RAAS suppression. However, some studies [32] still showed hemodynamic changes even with long-term use, suggesting preserved vascular response to epinephrine. No fatal cases were reported, even at high epinephrine concentrations (1:1000). However, severe adverse effects were noted [53,77,78], though not analyzed further in this review. At higher doses, the risk of interaction with non-selective β-blockers increases, suggesting a dose-dependent effect [21,58,59]. Nonetheless, this does not preclude the occurrence of such interactions at low doses, which can still trigger significant responses—as shown by conflicting outcomes in studies ([33] vs. [36]). Therefore, beyond dosage, factors like systemic absorption may influence the interaction [33]. Infiltration of epinephrine [33,39,40,41] in patients on non-selective β-blockers caused no major hemodynamic changes, while intravenous use at similar doses [32,35,36] led to significant hypertension and bradycardia. This discrepancy between studies using different administration routes suggests that the interaction may be more pronounced with intravenous epinephrine—such as during accidental intravascular injection (e.g., inferior alveolar nerve blocks). However, two case reports [53] described serious complications, including cardiac arrest, even after low-dose epinephrine infiltration into the eyelids in patients on long-term β-blocker therapy. According to what has been previously discussed, such outcomes would not have been expected. Therefore, it can be concluded that multiple factors could influence the body’s response to the interaction between epinephrine and β-blockers, including: dose of epinephrine, route of administration, duration of b-blocker administration, degree of systematic absorption from the injection site, adrenergic receptor sensitivity, vascularization of the anesthetized area, heart disease or other comorbidities, variation in vascular receptor response, or even idiosyncratic sensitivity of the peripheral vasculature to the alpha-pressor effect of epinephrine unmasked by the blockade of beta receptors [33].

In hypertensive patients on calcium channel blockers, epinephrine may enhance hypokalemia and increase the risk of arrhythmia [29,79,80]. This effect appears mainly with dihydropyridines, like nitrendipine, as shown in one study [42]. Epinephrine lowers plasma potassium via β_2_-receptor stimulation and activation of Na^+^/K^+^-ATPase. Nitrendipine may enhance this effect through several mechanisms: (a) upregulation or sensitization of β_2_-receptors, (b) direct activation of Na^+^/K^+^-ATPase, (c) inhibition of α-adrenergic-mediated potassium retention, (d) blockade of calcium-activated potassium channels, and (e) improved insulin sensitivity, promoting potassium shift into cells [42]. Two clinical studies [39,42] examined this interaction. One study [39] found a mild, non-significant drop in potassium with low-dose epinephrine in patients on long-term nifedipine and β-blockers—possibly due to nifedipine. The other study [42] reported no potassium change at low doses, but at higher epinephrine levels, nitrendipine enhanced hypokalemia (–0.5 mmol/L), without clinical concern. Overall, no significant interaction was seen with low-dose epinephrine.

Tolcapone and entacapone belong to the class of catechol-O-methyltransferase (COMT) inhibitors and act through reversible inhibition of the COMT enzyme. These agents have been incorporated into the pharmacological management of Parkinson’s disease as adjuncts to levodopa therapy, by preventing its breakdown [13,14,25,81]. COMT inhibitors may also interfere with the metabolism of exogenously administered epinephrine, since the COMT enzyme plays a direct role in epinephrine degradation [13,14,25,29,81,82]. Exogenous epinephrine is primarily inactivated by COMT in the liver [81]. If such an interaction occurs, the typically short duration of cardiovascular stimulation caused by epinephrine may be prolonged, as its vasoconstrictive effects are enhanced [13,18,81,82]. A human study [43] found no significant hemodynamic changes with a single dose of entacapone and low-dose epinephrine. However, at higher epinephrine doses [43], a significant heart rate increase and one case of ventricular tachycardia occurred, requiring propranolol for rhythm restoration.

Cocaine was introduced into medicine in 1884 as the first local anesthetic, and by 1903, it was combined with epinephrine to enhance its effectiveness. In 1924, this injectable combination was discontinued due to numerous reported deaths. Nevertheless, cocaine continues to be considered a useful drug because of its hemostatic and deep anesthetic properties and is still used today in combination with epinephrine, primarily in otolaryngologic procedures. However, cocaine today is predominantly associated with non-medical purposes, primarily through illicit recreational use [13,14,25,58,59]. Beyond its local anesthetic and strong vasoconstrictive properties, cocaine also acts as a central nervous system (CNS) stimulant [79]. Its sympathomimetic effects resemble those of tricyclic antidepressants, as it binds to neurotransmitter transporters and inhibits the reuptake of neurotransmitters into presynaptic neurons. This results in elevated levels of neurotransmitters in the synaptic cleft, leading to intensified postsynaptic receptor stimulation and pronounced vasoconstriction, which can cause hypertension and tachycardia [11,12,13,14,25,26,54,55,58,59,83,84]. Simultaneously, cocaine induces coronary artery vasoconstriction, reducing myocardial oxygen supply. The combination of increased myocardial oxygen demand and decreased coronary perfusion can result in myocardial ischemia, angina, or even myocardial infarction [11,25,26,54,55,59,83,84,85]. Cocaine, when combined with epinephrine, can lead to a potentially life-threatening interaction with unpredictable cardiovascular complications. These may include severe hypertensive crisis, serious arrhythmias, stroke or myocardial infarction, as well as severe morbidity or even death [11,13,20,25,54,58,59,61,73,82,83,84]. This interaction is mainly attributed to cocaine’s inhibition of neurotransmitter reuptake, a mechanism similar to that of tricyclic antidepressants, which enhances the effects of adrenergic vasoconstrictors [11,12,14,25,58,59,62,83,84,86]. Moreover, the blockade of cardiac muscarinic receptors may further enhance cardiovascular responses to administered vasoconstrictors [14,58,60]. Although there are many reports of serious, even fatal, complications from the combined use of cocaine and epinephrine, only one animal study [50] has examined low-dose epinephrine, suggesting that normal vascular reactivity may recover with continued exposure to cocaine, similar to what is seen with tricyclic antidepressants. Another study [87], using higher doses of epinephrine, found that the combined administration of epinephrine and 200 mg of cocaine did not significantly alter average blood pressure or heart rate and concluded that epinephrine is safe and effective when added to cocaine for nasal use, provided that cocaine doses do not exceed 200 mg. Additionally, one case report [88] described a pharmacologic interaction between high epinephrine dose and 2 mL of cocaine used as a combined local anesthetic, in which the patient experienced mild myocardial ischemia, presenting as intense chest tightness. Based on these findings, it appears that the co-administration of controlled doses of cocaine and epinephrine may not necessarily result in severe complications, particularly in cases of localized or medically supervised use. However, in dental settings, where the presence of cocaine is almost exclusively linked to illicit, unsupervised use, the exact dose and timing of intake are unknown. This makes risk assessment uncertain and the use of epinephrine potentially hazardous.

Digoxin is a cardiotonic glycoside used as second-line therapy for heart failure, typically after diuretics [11,14,25,26,89]. It has a narrow therapeutic index, making it prone to toxicity. One of its most serious risks is arrhythmia, which can increase the likelihood of acute myocardial infarction [14,25]. Epinephrine in local anesthetics has cardiotonic effects that may be enhanced when combined with digoxin, increasing the risk of serious arrhythmias [11,13,14,24,25,26,59,72,76,89]. Patients on digoxin often have underlying heart disease, making them more vulnerable to cardiovascular changes than the healthy subjects usually studied in dental clinical studies [14,25]. A single human study [44] found that low-dose infiltrated epinephrine in long-term digoxin users led to more frequent ECG changes—mainly asymptomatic tachycardia—compared to healthy controls. A similar study without epinephrine showed more and worse ECG changes, including arrhythmia, possibly due to differences in study design or patient characteristics [44].

Diuretics—especially non–potassium-sparing types—are commonly used for early hypertension and may interact with epinephrine by enhancing potassium loss. This additive effect can worsen epinephrine-induced hypokalemia, forming the basis of their interaction [21,29,39,46,62,73,75,79,80,82,90,91]. Epinephrine-induced hypokalemia is rapid and well documented, beginning within 10 min of injection [21,39,46,91,92]. It results from β2-receptor stimulation, which activates the Na^+^/K^+^-ATPase pump in skeletal muscle, shifting potassium intracellularly and lowering plasma levels [39,46,90]. Significant hypokalemia may lead to arrhythmias, as demonstrated in numerous studies [73,75,79,80,90]. However, the risk of arrhythmia appears to depend more on the rate of potassium decline rather than the absolute level of hypokalemia [39,46]. Some studies link even mild hypokalemia to fatal arrhythmias, while others argue that additional risk factors are typically needed [46]. Three clinical studies [39,45,46] found that non–potassium-sparing diuretics generally did not cause significant hemodynamic changes when combined with low-dose epinephrine, except for one reporting a drop in diastolic pressure [46]. However, all showed enhanced hypokalemia compared to epinephrine or diuretics alone, even with local infiltration of low doses, though it did not result in arrhythmia. A study using high-dose intravenous epinephrine [90] confirmed this effect without arrhythmias. While the potassium drop appears safe in healthy individuals, it may pose risks in patients with existing hypokalemia or those on digoxin, due to increased arrhythmia potential [46].

Monoamine oxidase inhibitors (MAOIs) were the first class of antidepressants used for treating depression [56,59]. Today, due to significant interactions with food and drugs they are limited only for those patients who do not respond to other types of antidepressants (tricyclics, SSRIs) [14,93,94,95,96,97,98,99]. MAOIs treat depression by inhibiting monoamine oxidase enzymes, which normally degrade neurotransmitters like serotonin, norepinephrine, and dopamine. This results in higher synaptic levels and enhanced receptor activity [54,56,59,93,94,95,96,98,99,100]. The interaction between MAOIs and epinephrine is mainly attributed to elevated levels of endogenous catecholamines caused by MAO inhibition. While exogenous epinephrine is metabolized by COMT, not MAO, both endogenous and exogenous catecholamines act on the same adrenergic receptors. This receptor overstimulation may lead to exaggerated cardiovascular responses [22,23,25,54,55,56,58,59,81,93,94,95,96,98,99,100,101,102]. MAOIs may also cause mild cardiac stimulation, especially at high doses, so the additive effects of epinephrine should be considered [24,81]. During early MAOI treatment, adrenergic receptors may be more sensitive to epinephrine due to lack of adaptation to increased neurotransmitters, potentially amplifying its effects [94,98]. In contrast, long-term MAOI use leads to receptor downregulation after 2–3 weeks, reducing the risk of strong cardiovascular responses [12,97,98]. Three studies—two in animals [8,51] and one in humans [47]—found no interaction between low-dose intravenous epinephrine and MAOIs, whether given short-term or acutely. Even with higher epinephrine doses [103], no significant hemodynamic changes were noted. These findings, along with the absence of case reports, suggest that clinically relevant interactions are unlikely at low doses.

Tricyclic antidepressants, once widely used for depression disorder, have largely been replaced by SSRIs due to their adverse effects and are now reserved for patients who do not tolerate or respond to newer treatments [13,28,58,60,93,94,95,96,104]. The primary mechanism by which tricyclic antidepressants exert their antidepressant and analgesic effects involves the inhibition of serotonin and norepinephrine reuptake into presynaptic neurons, through binding to their transport proteins, responsible for this process. As a result, increased concentration of endogenous neurotransmitters (serotonin and norepinephrine) is observed in the synaptic cleft, which are now free to interact more effectively with their postsynaptic receptors. This leads to neurochemical changes in brain that help alleviate symptoms of depression and pain [13,14,22,23,26,48,52,54,55,58,68,93,94,95,96,97,103,104]. Another mechanism of action, responsible for many of their adverse effects, involves antagonism of various receptors, such as histamine, acetylcholine, muscarinic, cholinergic, and α-adrenergic receptors [14,52,56,93,94,95,97,98,104,105]. The administration of exogenous epinephrine in the presence of elevated levels of endogenous catecholamines in the synaptic cleft—both acting on the same adrenergic receptors—may result in excessively high catecholamine concentrations and overstimulation of these receptors, potentially leading to hypertension and arrhythmias. Contrary to older views, the mechanism of interaction is not due to impaired epinephrine reuptake—since only endogenous catecholamines undergo neuronal reuptake, while exogenous epinephrine is metabolized by COMT [8,11,13,14,19,21,23,25,26,28,29,52,55,56,57,58,60,95,96,97,98,101,104,105,106]. Additionally, tricyclics may block α-receptors, shifting epinephrine’s action toward β2-mediated vasodilation, which can lead to hypotension, reflex tachycardia, reduced anesthesia duration, and increased risk of systemic toxicity [8,11,13,14,19,21,23,25,26,28,29,52,55,56,57,58,60,95,96,97,98,101,104,105,106]. Animal [8,45,52] and human [48] studies found no significant hemodynamic changes with low-dose epinephrine after short-term tricyclic antidepressant use, suggesting no confirmed interaction at typical dental doses. More serious effects like hypertension and arrhythmias were noted only with higher epinephrine doses [8,48,51,103], indicating a likely dose-dependent relationship. One animal study [52] showed no effects even at extremely high doses. No clinical case reports support this interaction at dental levels, though mild or asymptomatic cases may go unrecognized. Hypotension has not been reported even with high-dose epinephrine use [101]. Several studies suggest epinephrine causes fewer interactions than other vasoconstrictors in patients on tricyclic antidepressants [22,23,54,58,61,62,81,101,107,108,109], making it the preferred choice when a vasoconstrictor is needed [99]. Long-term tricyclic use leads to β-receptor downregulation starting around 2–3 weeks, potentially reducing sensitivity to epinephrine and lowering cardiovascular risk. Thus, patients on short-term therapy may be more reactive to epinephrine [14,21,25,57,58,60,98,101].

Other drugs have been theoretically associated with epinephrine interactions based on their pharmacological profile. However, these have not been confirmed by clinical data or case reports to date. One such example is serotonin and norepinephrine reuptake inhibitors (SNRIs), a newer class of antidepressants. They inhibit the reuptake of these neurotransmitters, increasing their levels in the synaptic cleft—something that could enhance the cardiovascular effects of vasoconstrictors when administered concurrently [57,98]. Another drug category, adrenergic neuron blocking agents, such as guanethidine and reserpine, lowers blood pressure by inhibiting norepinephrine release from sympathetic nerve terminals. Prolonged use may upregulate postsynaptic adrenergic receptors, increasing sensitivity to vasoconstrictors like epinephrine and potentially enhancing systemic responses [13,14,23,25,58,60,69,70,76]. Clonidine, a central α2-agonist used for hypertension, also partially stimulates peripheral α2-receptors, potentially interacting with epinephrine, which also acts on postsynaptic peripheral α2-adrenergic receptors. This may alter epinephrine’s vasoconstrictive effect—either reducing local anesthesia efficacy or, if systemically absorbed, enhancing cardiovascular responses. Chronic use may further increase sensitivity to vasoconstrictors due to adrenergic receptor upregulation [57,70,73,76,82,105]. Nitrates like nitroglycerin, used for angina and hypertension, may enhance systemic responses to epinephrine due to increased adrenergic receptor sensitivity. As vasodilators, they can also reduce epinephrine’s local vasoconstrictive effect, possibly shortening the duration of anesthesia [18,26,76,89]. Adrenoreceptor agonists like ephedrine and orciprenaline may amplify epinephrine’s systemic effects. Ephedrine, acting directly on α1, β1, and β2 receptors and indirectly by releasing norepinephrine, can potentiate epinephrine’s cardiovascular actions. Orciprenaline, a β2-agonist bronchodilator, may enhance epinephrine-induced hypokalemia due to additive β2-stimulation, especially in susceptible patients [62]. Stimulant laxatives like bisacodyl do not directly interact with epinephrine pharmacologically. However, they may indirectly increase the risk of epinephrine-induced hypokalemia by causing potassium loss, especially with prolonged use. This combined effect may heighten the risk of arrhythmias in patients with existing or severe hypokalemia [62]. Central nervous system (CNS) stimulants will be discussed separately from cocaine, as these medications are typically administered in controlled, low doses, mainly for the treatment of Attention deficit hyperactivity disorder (ADHD) in children and adults [25,57]. Amphetamine and its derivatives (e.g., dextroamphetamine), as well as other CNS stimulants, such as methylphenidate, increase norepinephrine and dopamine levels in the brain by promoting their release and inhibiting their reuptake, stimulating the adrenergic system. These agents can raise blood pressure and heart rate, and in rare cases, have been linked to serious cardiovascular events. When combined with epinephrine-containing local anesthetics, additive adrenergic effects may elevate the risk of hypertension, stroke, or cardiac complications—especially in patients with underlying heart disease. Abuse of methamphetamine or amphetamine-based drugs further increases these risks. Epinephrine use is contraindicated within 24 h of methamphetamine use, with some guidelines suggesting a 12 h minimum wait. For such patients, anesthetics without epinephrine are considered safer [11,24,25,29,57,59,60,62,105,110]. Another drug associated with potential interactions is atomoxetine, which is primarily used for the treatment of Attention deficit hyperactivity disorder (ADHD) in children and adults. Unlike typical stimulant medications, atomoxetine is not a central nervous system stimulant. Pharmacologically, it shares similarities with tricyclic antidepressants due to its similar mechanism of action on norepinephrine reuptake [25,57]. Atomoxetine is a selective norepinephrine reuptake inhibitor (NRI), which increases norepinephrine levels in the synaptic cleft by blocking its reuptake into the presynaptic neuron. The elevation of norepinephrine levels both in the central nervous system and peripherally may lead to cardiovascular effects, such as increased blood pressure and heart rate—phenomena also observed with stimulant medications. The combination of atomoxetine with vasoconstrictors used in dental local anesthetics may have an additive effect on adrenergic activity, potentially intensifying cardiovascular responses [57,105]. Finally, among halogenated inhalational anesthetics, most (e.g., halothane, fluroxene, methoxyflurane) are no longer used due to adverse effects. Isoflurane remains in limited use, while enflurane is nearly obsolete [14,58,59,60,111]. These agents may enhance the arrhythmogenic effects of catecholamines, particularly by increasing β1-receptor sensitivity in the heart and α1-receptor sensitivity in the vasculature, potentially leading to exaggerated cardiovascular responses and arrhythmias [11,12,13,14,20,29,62,69,112]. Halothane is the most strongly associated with this interaction, with several reports of severe arrhythmias when combined with epinephrine [13,14,22,58]. Other agents (enflurane, isoflurane, etc.) have minimal effect on myocardial sensitivity to epinephrine [22,23]. Notably, most studies [113,114,115,116] demonstrating interactions used high epinephrine doses, which exceed typical dental or minor surgical use. A fatal case [117] has been reported involving a patient under halothane anesthesia who received a gingival retraction cord with epinephrine.

Regarding the second part of the present review, a classification of the pharmacological interactions was conducted, based on the criteria of the UpToDate Lexidrug platform, as presented in Table 8. However, when this classification is compared with that provided by the platform for each individual drug (see Table 9), several discrepancies are observed. It should be noted that the classification in Table 8 was based exclusively on the use of low-dose epinephrine, as it is applied locally in routine dental practice. In contrast, the classification used by the UpToDate Lexidrug platform refers to the general (systemic) use of epinephrine, without specifying the dose or route of administration. Specifically, the search in the platform was performed using the term “Epinephrine (Systemic)”. Consequently, the differences between the two classifications may be attributed both to the variation in dosage and to the overall risk profile associated with systemic administration of epinephrine. In any case, the information referring to the general use of epinephrine cannot be directly applied to scenarios involving low, locally administered doses, as commonly used in dental practice. Therefore, the risk assessment may differ significantly.

## 5. Limitations

However, this review has certain limitations. As shown by the included studies, available data on drug interactions with epinephrine remain limited. Clinical studies are few, often involving small sample sizes, while findings from animal models cannot always be reliably extrapolated to humans. Moreover, the variability in study design and methodology further complicates data interpretation. The lack of documented adverse events in dental settings—particularly concerning long-standing medications—may suggest that serious interactions are rare; however, such symptoms may sometimes go unrecognized or misattributed to anxiety. In addition, the absence of recent research highlights the need for updated, large-scale clinical investigations. Future research should prioritize well-designed, prospective clinical studies with sufficient sample sizes to better clarify potential interactions between epinephrine and commonly prescribed medications. Establishing clinical registries in dental and hospital settings could also help capture adverse events that may go unreported in everyday practice, enhancing epidemiological insight. Additionally, pharmacokinetic and pharmacodynamic studies in patients with comorbidities and polypharmacy are essential for understanding interaction mechanisms and individual variability.

## 6. Conclusions

The following clinical implications and recommendations are particularly important for safe dental practice. Ensuring adequate local anesthesia is essential, as insufficient pain control can trigger a surge of endogenous epinephrine exceeding the cardiovascular impact of the small exogenous dose [118]. Dentists should always perform aspiration testing and inject the anesthetic slowly to minimize the risk of intravascular administration [119,120,121]. The minimal effective dose should be used; up to three cartridges of epinephrine 1:100,000 or 1:80,000 is generally considered safe, while higher doses may increase the likelihood of dose-dependent interactions, as seen in this study. Regular monitoring of the patient’s vital signs before and during dental treatment is strongly recommended [122]. A thorough medical history is crucial, as drug interactions can be clinically significant in patients with underlying cardiovascular disease [9]. Particular caution is advised at the beginning of a new pharmacological therapy, when the patient’s body has not yet adapted to the medication’s effects [31,35,98]. Whenever feasible, articaine is preferred for infiltration anesthesia over block injections to further reduce systemic exposure [119]. Ultimately, the establishment of standardized clinical guidelines based on robust evidence could greatly enhance the safety of dental procedures, especially for patients at increased cardiovascular or pharmacological risk.

## Figures and Tables

**Table 1:** Severity level.

Severity Level	Description
Major	The interaction may be life-threatening or require medical intervention to minimize or prevent serious adverse effects or life-threatening or debilitating long-term (e.g., >1 year) sequelae. The loss of therapeutic efficacy of an agent used for potentially life-threatening conditions or an increase in the risk of unintended pregnancy may also be considered major.
Moderate	The interaction may alter a patient’s clinical condition in a way that is likely to cause significant adverse clinical outcomes or interfere with medical care (e.g., by altering diagnostic results) but does not pose a clear risk of outcomes associated with the major category.
Minor	The interaction may cause unwanted effects but does not pose a clear risk of negative outcomes associated with moderate or major categories. Interactions with this rating are unlikely to require additional or extended clinical care or to substantially alter outcomes.

**Table 2:** Reliability Rating.

Reliability Rating	Description
Highest	Documented by 2 or more well-conducted, well-controlled human studies. Evidence supporting the interaction greatly outweighs evidence against the interaction.
Intermediate-Hight	Interaction is supported by at least 1 well-conducted, well-controlled human study and at least 5 cases of other lower quality studies. Evidence for an interaction greatly outweighs evidence against an interaction.
Intermediate	Documented by at least 1 well-conducted, well-controlled human study, at least 3 case reports, at least 2 studies that do not qualify as well-conducted and well-controlled human studies, or a combination of these. Studies and cases may come from official product labeling or published literature
Intermediate-Low	Plausible interaction based on the known pharmacology of the agents, meeting 1 of the following criteria, and where the evidence supporting an interaction outweighs evidence against an interaction: At least 2 case reports; a single study that does not qualify as a well-conducted, well-controlled human study; or a combination of these. Evidence that would otherwise qualify for a higher rating but for which there are substantial contradictory results or reports. Product labeling statement supported by either no data or by data that would not qualify for a higher rating
Lowest	Potential interaction meeting 1 or more of the following criteria: A single case report with questionable mechanistic basis. Theoretical without sound mechanistic or clinical support. Evidence of no interaction greatly outweighs evidence supporting an interaction.

**Table 3:** Risk rating.

Risk Rating	Action	Description
A	Not known interaction	Data have not demonstrated either pharmacodynamic or pharmacokinetic interactions between the specified agents.
B	No action needed	Data demonstrate that the specified agents may interact with each other, but there is little to no evidence of clinical concern resulting from their concomitant use.
C	Monitor	Data demonstrate that the specified agents may interact with each other in a clinically significant manner. The benefits of concomitant use of these two medications often outweigh the risks. An appropriate monitoring plan should be implemented to identify potential negative effects. Dosage adjustments of one or both agents may be needed in some patients.
D	Consider therapy modification	Data demonstrate that the two medications may interact with each other in a clinically significant manner. A patient-specific assessment must be conducted to determine whether the benefits of concomitant therapy outweigh the risks. Specific actions must be taken in order to realize the benefits and/or minimize the risks resulting from concomitant use of the agents. These actions may include aggressive monitoring, empiric dosage changes, or choosing alternative agents.
X	Avoid	Data demonstrate that the specified agents may interact with each other in a clinically significant manner. The risks associated with concomitant use of these agents usually outweigh the benefits. Concurrent use of these agents should generally be avoided.

**Table 4:** Clinical studies involving human subjects.

Drug Class	Author(s)/Year of Publication	Sample Size/Health Status	Interacting Drug/Treatment Duration	Dose of Epinephrine/Administration Route	Results
Antipsychotics	Shionoya et al., 2020 [31]	- *n* = 30 - under general anesthesia with propofol	- typical or atypical - more than 3 months	- intraoral infiltration - 22.5 μg E 1:80,000	- SBP, DBP, HR, SpO_2_: no significant changes 10 and 1 h after E administration
B-blockers	Houben et al., 1982 [32]	- *n* = 5 - hypertension	- propranolol - (non-selective), or metoprolol - (selective) - long-term use with a 4-week drug crossover	- intravenous infusion (0.5/1/2/4 μg/min, ×8′) - equivalent doses: 4, +8, +16, +32 µg Ε (total dose 60 μg)	- Propranolol: significant increase in DBP (+5 mmHg) and significant decrease in HR (−3 bpm) even at the lowest dose (4 μg E), along with a significant increase in SBP (+33 mmHg) at the maximum dose (60 μg E). - Metoprolol: no significant changes
	Dzubow, 1986 [33]	- *n* = 10 - hypertension	- propranolol (non-selective) - long-term use	- local skin injection - 10–60 μg Ε (mean dose: 30 μg)	- MAP decreased (−0.4 ± 3.4 mmHg) 5–15′ after E - The difference was statistically significant, when compared with another group with 10 healthy subjects without propranolol (MAP: −8.9 ± 12.4 mmHg 5–15′ after E)
	Leenen et al., 1988 [34]	- *n* = 7 - healthy subjects	- atenolol (selective), or propranolol (non-selective), or placebo - single oral dose (1.5 h before E)	- intravenous infusion (10/20/40/80 ng/kg/ min, ×6′) - equivalent dose: 4.59 μg, +9.18 μg, +18.36 μg, +36.72 μg, for a 76.5 kg patient	- Only changes up to 40 ng/kg/min (total dose 32.13 μg over 18 min) will be discussed. - Atenolol/E: significantly lower SBP at 40 ng/kg/min compared to placebo, and significantly lower DBP (from 20 ng/kg/min onward) compared to both propranolol and placebo. - Propranolol/E: significantly higher DBP and significantly lower HR (from 10 ng/kg/min) compared to placebo. HR was also significantly lower than in the atenolol/E group at 20 and 40 ng/kg/min. The P/V index remained stable with propranolol, but was lower than placebo at 40 ng/kg/min.
	Ichinohe et al., 1991 [35]	- *n* = 6 - healthy subjects	- propranolol (non-selective) - single intravenous infusion (15′ before E)	- intravenous infusion (10 ng/kg/ min, ×15′) - equivalent dose: 10.5 μg, for a 70 kg patient	- Significant decrease in HR (−5.6 ± 3.2%) and CI (−12.7 ± 12.1%), and significant increase in MAP (+15.1 ± 14.1%) and TPR (+36.4 ± 34.6%), after E administration - Ιncreased cardiac pressure work was observed, indicating elevated myocardial oxygen demand
	Mackie & Lam, 1991 [36]	- *n* = 6 - healthy subjects	- propranolol (non-selective) - single intravenous infusion (5′ before E)	- intravenous infusion - 15 μg Ε	- Significant hypertension (MAP: +12 mmHg) and bradycardia (HR: −18 bpm) after E. - Clinical symptoms: 2 cases of arrhythmia and 1 case of chest tightness (with unclear resolution).
	Sugimura et al., 1995 [37]	- *n* = 6 - healthy subjects	- pindolol (non-selective) - single oral dose (1 h before anesthesia)	- intraoral infiltration - 45 μg E 1:80,000, and later local anesthesia without Ε	- CO: significant lower in the presence of E, compared to the absence of E (3.8 ± 0.5 vs. 4.9 ± 0.6 c/min at 5 min, and 4.0 ± 0.4 c/min vs. 4.8 ± 0.7 c/min at 10 min). - E anesthesia also caused significant reduction in TPR and decreased myocardial contractility at 5–10′, compared to anesthesia without E.
	Niwa et al., 1996 [38]	- *n* = 7 - healthy subjects	- pindolol (non-selective) and repetition of the experiment without pindolol - single oral dose (1 h before E)	- intraoral infiltration - 45 μg Ε 1:80,000	- Pindolol/E: significant increase in SBP, compared to E alone (+8% vs. 0%), at 2′. - DBP: increased (+12% at 2′/+10% at 5′) in group with pindolol/E, whereas in group with E alone, it remained unchanged (no significant difference between the two groups) - According to Doppler findings, E alone improved left ventricular relaxation (diastolic function), whereas in the pindolol/E group, relaxation was delayed.
	Meechan, 1997 [39]	- *n* = 6 - hypertension	- non-selective - long-term use	- intraoral infiltration - 55 μg Ε 1:80,000	- BP: no significant change, 10′ after E - HR: mild decrease, 10′ after E
	Zhang et al., 1999 [40]	- *n* = 10 - healthy subjects	- nadolol (non-selective) and repetition of the experiment with placebo - single oral dose, (3 h before E)	- intraoral infiltration - 10 μg Ε 1:100,000, or anesthesia without Ε	- Νadolol/E: significantly prolonged duration of pulpal anesthesia (+17′) and soft tissue anesthesia (+16.5′) compared to the placebo/E - No difference in anesthesia duration was observed between nadolol and placebo in the absence of E. - No significant changes in BP or HR were noted with nadolol/E. - Clinical symptoms included fatigue in 3 individuals (2 on nadolol, 1 on placebo) and transient gum sensitivity in 2 individuals (group not specified).
	Niwa et al., 2001 [41]	- *n* = 12 - heart disease	- selective (*n* = 9) and non-selective (*n* = 3) - long-term use	- intraoral infiltration - 22 μg Ε 1:80,000	- Non-selective: mild increase in MAP, SV, and TPR, and mild decrease in CI, after E. - Non-selective: significantly greater increases in MAP (+13.2% vs. 0%) and TPR (+11% vs. −11%) at 2′ after E, compared to selective. - Non-selective: significantly higher MAP at 2′, elevated TPR from 2′ to 10′, and significantly lower CI at 5′ after E, compared to 15 patients on other types of anti-hypertensives
Calcium channel blockers	Mimran et al., 1993 [42]	- *n* = 31 - healthy subjects	- nitrendipine (*n* = 16), verapamil (*n* = 7), diltiazem (*n* = 8), and repetition of the experiment with placebo - for 4 days, before E	- intravenous infusion (12.5/25/50 ng/kg/min, ×30′) - equivalent doses: 26, +52, +105 μg, for a 70 kg patient	- At a low dose of E (26 μg), none of the calcium channel blockers affect K^+^ levels compared to placebo. High doses beyond the approved limits were not investigated. - Only diltiazem significantly reduces glucose levels (−0.5 mmol/L) compared to placebo, without clinical manifestations.
	Meechan, 1997 [39]	- *n* = 6 - hypertension	- (*n* = 2) nifedipine - long-term use	- intraoral infiltration - 55 μg Ε 1:80,000	- Slight decrease in K^+^ (−0.03 mmol/L), 10′ after E, in 3 out of 6 participants—possibly attributed to nifedipine.
Catechol-O-methyltransferase inhibitors	Illi et al., 1995 [43]	- *n* = 5 - healthy subjects	- entacapone - single dose (30′ before E)	- intravenous infusion (1.5/3/6/12 μg/min, ×5′) - equivalent doses: 7.5 μg, +15 μg, +30 μg, +60 μg	- At 15’ (total dose 52.5 μg), there was a mild increase in SBP and HR, and a decrease in DBP compared to baseline values, with no significant differences from other 6 individuals on placebo. - High doses beyond the approved limits were not investigated (15′ after).
Digitalis glycosides	Blinder et al., 1998 [44]	- *n* = 14 - heart disease	- digoxin - long-term use	- intraoral infiltration - 54 μg Ε 1:100,000	- A higher frequency of ECG changes was observed 2 h after E in participants on digoxin, compared to 26 healthy individuals not on digoxin, without clinical manifestations. - E increased tachycardia but reduced arrhythmias and myocardial ischemia (compared to another study without E).
Diuretics	Whyte et al., 1988 [45]	- *n* = 8 - healthy subjects	- spironolactone - (potassium sparing) or bendroflumethiazide, furosemide (non-potassium sparing) - for 14 days, before E	- intravenous infusion - (0.01/0.02/0.06 μg/kg/min, (×10’ each), then maintained at 0.06 μg/kg/min, ×90′)	- SBP, DBP: no differences up to 20’, during which the E dose was low (a total of 21 μg for a 70 kg patient) - Mild decrease in K^+^ and Mg^2+^ and a slight increase in HR, across all three types of diuretics. - The maximum effect, beyond the study’s limits, was not investigated.
	Meechan & Rawlins, 1992 [46]	- *n* = 8 - hypertension	- non–potassium-sparing - more than 1 year	- intraoral infiltration - 55 μg Ε 1:80,000	- SBP, HR: no significant change, 10’ after E. - A significant decrease in K^+^ (up to −0.6 mmol/L, with a minimum of 2.6 mmol/L) and in DBP (−14 mmHg), at 10’. - 50% of participants had K^+^ levels below the normal range at 10′, but without clinical manifestations.
	Meechan, 1997 [39]	- *n* = 8 - hypertension	- non–potassium-sparing - long-term use	- intraoral infiltration - 55 μg Ε 1:80,000	- No significant change in BP, mild increase in HR, and marked decrease in K^+^ (up to −0.06 mmol/L, with a minimum of 2.6 mmol/L), 10′ after E. - 50% of participants had K^+^ levels below the normal range, but without clinical manifestations.
Monoamine oxidase inhibitor	Cuthbert & Vere, 1971 [47]	- *n* = 3 - healthy subjects	- tranylcypromine - 8–14 days, before E	- intravenous infusion (simulation of inadvertent intravenous injection) - small doses 1–36 μg	- A moderate enhancement (2–4 fold) of E action was observed on HR (increase) and DBP (decrease), with a lesser impact on SBP (increase).
Tricyclic antidepressants	Svedmyr, 1968 [48]	- *n* = 2 - healthy subjects	- protriptyline - 4 days, before E	- intravenous infusion (0.0074/0.022/0.067/0.20 μg/kg/ min, ×10′) before protriptyline - intravenous infusion (0.00082/0.0025/0.007/0.022/0.067 μg/kg/min, ×10′), after protriptyline	- With protriptyline, only one-third of the E dose was required to produce the same hemodynamic changes (increased SBP, DBP, and HR) as E alone. - Hemodynamic changes began at a dose of 0.022 μg/kg/min (total dose up to 40 min = 23 μg for a 70 kg patient) in the E/protriptyline group. Comparable changes with E alone required a threefold higher dose of 0.067 μg/kg/min (total dose up to 30 min = 67.5 μg for a 70 kg patient). - The maximum effect, beyond the study’s scope, was not investigated.

Abbreviations: SBP = Systolic Blood Pressure, DBP = Diastolic Blood Pressure, HR = Heart Rate, SpO_2_ = oxygen saturation, Ε = epinephrine, BP = Blood Pressure, K^+^ = Potassium, Mg^2+^ = Magnesium, CI = Cardiac Index, ECG = Electrocardiogram, TPR = ΣΠA= Total Peripheral Resistance, P/V index = Index of myocardial contractility, CO = Cardiac Output, MAP = Mean Arterial Blood Pressure, SV = Stroke Volume.

**Table 5:** Animal experimental studies.

Drug Class	Author(s)/Year of Publication	Sample Size/Health Status	Interacting Drug/Treatment Duration	Dose of Epinephrine/Administration Route	Results
Antipsychotics	Yagiela et al., 1985 [8]	- *n* = 4 dogs - no other medications	- chlorpromazine - single dose (1 h before E)	- intravenous infusion 0.33–2.5 μg/kg - 0.33 μg/kg ≈ 1 cartridge (1:100,000 E) in 55 kg female, while 2.5 μg/kg ≈ 7.5 cartridges	- At low doses of E, no interaction with chlorpromazine was observed (no changes in BP, HR, or occurrence of arrhythmias).
	Higuchi et al., 2014 [49]	- *n* = 6 rats - no other medications	- chlorpromazine - single dose (20′ before E)	- intraperitoneal injection - doses of 1, 10, and 100 μg/kg	- At low doses (1 μg/kg E), no significant hemodynamic changes (mild hypotension: 99.0 ± 18.6% and mild tachycardia: 99.6 ± 6.4%). - Higher doses were not investigated.
Cocaine	Bernards et al., 1997 [50]	- *n* = 6 sheep - no other medications	- administered intravenously in a human-like pattern - for 18 days before E	- intravenous infusion - 0.15 μg/kg E	- The effect of E was not altered by cocaine when administered 15 and 18 days after cocaine use (values remained the same before and after cocaine).
Monoamine oxidase inhibitors	Wong et al., 1980 [51]	- *n* = 6 dogs - under general anesthesia with halothane, enflurane, or methoxyflurane	- pargyline - for 8–14 days before E	- intravenous infusion - 1 μg/kg/min E until the occurrence of ≥3 consecutive dysrhythmic events (arrhythmia precursor)	- Pargyline did not significantly alter sensitivity to E (the E dose that induced arrhythmia was >3.6–4.2 μg/kg in dogs without MAOI, and only slightly higher in dogs treated with MAOI). - BP remained unchanged. - These doses are far above those used in dentistry, suggesting that lower doses are unlikely to induce arrhythmias.
	Yagiela et al., 1985 [8]	- *n* = 4 dogs - no other medications	- phenelzine - single dose (1 h before E)	- intravenous infusion 0.33–2.5 μg/kg - 0.33 μg/kg ≈ 1 cartridge (1:100,000 E) in 55 kg female, while 2.5 μg/kg ≈ 7.5 cartridges	- No significant effect was observed on any of the studied parameters (BP, HR).
Tricyclic antidepressants	Wong et al., 1980 [51]	- *n* = 6 dogs - under general anesthesia with halothane, enflurane, or methoxyflurane	- imipramine - for 8–14 days, before E	- intravenous infusion - 1 μg/kg/min E until the occurrence of ≥3 consecutive dysrhythmic events (arrhythmia precursor)	- Imipramine significantly reduced the E dose required to induce arrhythmia (from 3.6–4.2 μg/kg to 1.43–3.23 μg/kg), particularly under halothane and enflurane anesthesia. - BP remained unchanged. - These doses are much higher than those used in dentistry, suggesting that lower doses are unlikely to cause arrhythmias.
	Yagiela et al., 1985 [8]	- *n* = 4 dogs - no other medications	- desipramine - single dose (1 h before E)	- intravenous infusion 0.33–2.5 μg/kg - 0.33 μg/kg ≈ 1 cartridge (1:100,000 E) in 55 kg female, while 2.5 μg/kg ≈ 7.5 cartridges	- At the lowest E dose (0.33 μg/kg), no changes in MAP or HR were observed, and no arrhythmias occurred. - Higher doses were not investigated.
	Oliveira et al., 2022 [52]	- *n* = 7 rats - no other medications	- amitriptyline, at a dose equivalent to that used in a 70 kg human - for 7 days, before E	- intravenous infusion and intraosseous infiltration - doses of 160, 640, and 2560 ng E (=2, 8, 32 cartridges of 1:100,000 E)	- Mild increase in BP was observed even at high doses of E after infiltration, both with and without amitriptyline. - A significant increase in BP occurred after intravenous infusion, even at the low dose, with and without amitriptyline. - Therefore, the antidepressant does not influence the BP increase induced by E.

Abbreviations: HR = Heart Rate, Ε = Epinephrine, BP = Blood Pressure, MAP = Mean Arterial Blood Pressure.

**Table 6:** Case reports.

Drug Class	Author(s)/Year of Publication	Age/Gender/Medical History	Interacting Drug/Treatment Duration	Dose of Epinephrine/Administration Route	Results
B-blockers	Foster & Aston, 1983 [53]	- 52 years old - female - hypertension	- propranolol (non-selective) - 40 mg, twice daily - long-term use	- 13 mL of 0.5% lidocaine solution, 1:200,000 E - total dose: 65 μg E - injected into the upper eyelids and upper neck region for plastic surgery	- Severe hypertension (200/110 mmHg) and subsequent cardiac arrest occurred 60’after E. - The patient was intubated, received CPR, and ultimately survived.
	Foster & Aston, 1983 [53]	- 58 years old - male - hypertension	- propranolol (non-selective) - 20 mg, three times daily - long-term use	- 8 mL of 0.5% lidocaine solution, 1:200,000 E - total dose: 40 μg E - used for blepharoplasty surgery	- Severe hypertension (from 120/80 mmHg to 260/150 mmHg) and bradycardia (from 60 bpm to 52 bpm) after E. - The patient returned to baseline (BP = 130/80 mmHg and HR = 58 bpm), 10′ after administration of 20 mg hydralazine. - The surgery continued without further complications.

Abbreviations: E = epinephrine, BP = Blood Pressure, HR = Heart Rate, CPR = Cardiopulmonary Resuscitation.

**Table 7:** Generic and Commercial (Brand) Names of Drugs Included in the Review.

Generic Name	Commercial (Brand) Name
Chlorpromazine	Solidon^®^, Thorazine^®^, Largactil^®^
Propranolol	Inderal^®^, Hemangeol^®^
Metoprolol	Lopressor^®^
Atenolol	Tenormin^®^
Pindolol	Visken^®^
Nadolol	Corgard^®^
Nifedipine	Adalat^®^, Procardia^®^
Nitrendipine	Baypress^®^, Nitrel^®^, Nifecard^®^, Nitrendilate^®^
Entacapone	Comtan^®^
Digoxin	Lanoxin^®^
Spironolactone	Aldactone^®^
Bendroflumethiazide	Naturetin^®^
Furosemide	Lasix^®^
Tranylcypromine	Parnate^®^
Pargyline	Eutonyl^®^
Phenelzine	Nardil^®^
Imipramine	Tofranil^®^
Desipramine	Norpramin^®^
Amitriptyline	Elavil^®^
Protriptyline	Vivactil^®^

**Table 8:** Classification of Low-Dose Epinephrine Interactions Based on Lexidrug Criteria and Literature Data.

Interacting Drug	Severity	Reliability Rating	Risk Rating
Antipsychotics	Minor	Intermediate–Low	B
B-blockers (non-selective)	Major	Intermediate	D
Calcium channel blockers	Minor	Intermediate	B
COMT Inhibitors	Minor	Intermediate–Low	B
Cocaine	Major	Lowest	D
Digitalis Glycosides	Moderate	Intermediate–Low	C
Diuretics (non-potassium-sparing)	Moderate	Intermediate	D
Monoamine Oxidase Inhibitors	Minor	Intermediate–Low	B
Tricyclic Antidepressants	Minor	Intermediate–Low	B

**Table 9:** Lexidrug-Based Classification of Systemic Epinephrine Interactions.

Interacting Drug	Severity	Reliability Rating	Risk Rating
Antipsychotics	Major	Lowest	D
B-blockers (non-selective)	Moderate	Highest	C
Calcium channel blockers	No interactions of Risk Level A or greater identified		
COMT Inhibitors	Moderate	Intermediate	C
Cocaine	Major	Intermediate	D
Digitalis Glycosides	Moderate	Intermediate–Low	C
Diuretics (non–potassium sparing)	Moderate	Intermediate–Low	C
Monoamine Oxidase Inhibitors	Moderate	Intermediate–Low	C
Tricyclic Antidepressants	Moderate	Intermediate–Low	D

## Data Availability

Not applicable.
